# Essential Microenvironment for Thymopoiesis is Preserved in Human Adult and Aged Thymus

**DOI:** 10.1080/10446670310001598465

**Published:** 2003-03

**Authors:** J. Shiraishi, M. Utsuyama, S. Seki, H. Akamatsu, M. Sunamori, M. Kasai, K. Hirokawa

**Affiliations:** 1 Department of Pathology and Immunology Tokyo Medical and Dental University Graduate School 1-5-45, Yushima, Bunkyo-ku Tokyo 113-8519 Japan; 2 Department of Cardiothoracic Surgery Tokyo Medical and Dental University Graduate School 1-5-45, Yushima, Bunkyo-ku Tokyo 113-8519 Japan; 3 Department of Bacterial and Blood Products National Institute of Infectious Diseases 1-23-1, Toyama, Shinjuku-ku Tokyo 162-8640 Japan

## Abstract

Normal human thymuses at various ages were immunohistologically examined in order to determine whether adult or aged thymus maintained the microenvironment for the T cell development and thymopoiesis was really ongoing. To analyze the thymic microenvironment, two monoclonal antibodies (MoAb) were employed. One is MoAb to IL-1 receptor (IL-1R) recognizing medullary and subcapsular cortical epithelial cells of normal infant human thymus. The other is UH-1 MoAb recognizing thymic epithelial cells within the cortex, which are negative with IL-1R-MoAb. Thymus of subjects over 20 years of age was split into many fragments and dispersed in the fatty tissue. However, the microenvironment of each fragment was composed of both IL-1R positive and UH-1 positive epithelial cells, and the UH-1 positive portion was populated with lymphocytes showing a follicle-like appearance. Lymphocytes in these follicle-like portions were mostly CD4^+^CD8^+^ double positive cells and contained many proliferating cells as well as apoptotic cells. Thus these follicle-like portions in adult and aged thymus were considered to be functioning as cortex as in infant thymus. Proliferative activity of thymocytes in the thymic cortex and the follicle-like portions definitely declined with advance of age, while incidence of apoptotic thymocytes increased with aging.


*Clinical & Developmental Immunology*, March 2003, Vol. 10 (1), pp. 53–59

Taylor & Francis healthsciences

### Essential Microenvironment for Thymopoiesis is Preserved in Human Adult and Aged Thymus

J. SHIRAISHI[a], M. UTSUYAMA[a], S. SEKI[a], H. AKAMATSU[b], M. SUNAMORI[b], M. KASAI[c] and K. HIROKAWA[a,*]

[a]*Department of Pathology and Immunology, Tokyo Medical and Dental University Graduate School, 1-5-45, Yushima, Bunkyo-ku, Tokyo 113-8519, Japan;* [b]*Department of Cardiothoracic Surgery, Tokyo Medical and Dental University Graduate School, 1-5-45, Yushima, Bunkyo-ku, Tokyo 113-8519, Japan;* [c]*Department of Bacterial and Blood Products, National Institute of Infectious Diseases, 1-23-1, Toyama, Shinjuku-ku, Tokyo 162-8640, Japan*

Normal human thymuses at various ages were immunohistologically examined in order to determine whether adult or aged thymus maintained the microenvironment for the T cell development and thymopoiesis was really ongoing. To analyze the thymic microenvironment, two monoclonal antibodies (MoAb) were employed. One is MoAb to IL-1 receptor (IL-1R) recognizing medullary and subcapsular cortical epithelial cells of normal infant human thymus. The other is UH-1 MoAb recognizing thymic epithelial cells within the cortex, which are negative with IL-1R-MoAb. Thymus of subjects over 20 years of age was split into many fragments and dispersed in the fatty tissue. However, the microenvironment of each fragment was composed of both IL-1R positive and UH-1 positive epithelial cells, and the UH-1 positive portion was populated with lymphocytes showing a follicle-like appearance. Lymphocytes in these follicle-like portions were mostly $CD4^+CD8^+$ double positive cells and contained many proliferating cells as well as apoptotic cells. Thus these follicle-like portions in adult and aged thymus were considered to be functioning as cortex as in infant thymus. Proliferative activity of thymocytes in the thymic cortex and the follicle-like portions definitely declined with advance of age, while incidence of apoptotic thymocytes increased with aging.

*Keywords*: Aging; Thymus; Apoptosis; Lymphocytes; Microenvironment

### INTRODUCTION

The thymus is an important organ for the development of T cell dependent immune system. Such an important thymus, however, is known to start to involute around at puberty and its function is believed to become marginal in the late adult and the elderly.

Using mouse model, we reported that the thymic function started to decline in the early phase of the life (Hirokawa and Makinodan, 1975; Hirokawa *et al.*, 1988a,b; Hirokawa, 1998). Thus, the abilities of mouse thymus at 4 weeks of age showed a distinct decline as compared with those of the newborn mice; i.e. immigration of thymocytes precursors into the thymus dropped to 6%, proliferation of thymocytes to 80% and emigration of mature T cells to the periphery to 7%, as compared with newborn mice (Hirokawa *et al.*, 1994).

The early onset of decline of thymic function was also reported in human. The thymic epithelial cells as core element of thymic tissue starts to reduce in volume as early as one year of age (Steinmann *et al.*, 1985). MacKall *et al.* (1995) examined regeneration of $CD4^+$ T cells in cancer patients after the intensive chemotherapy and found that regeneration of $CD4^+$ T cells in the peripheral blood was very retarded in the young adult patients as compared with infant patients. They suggested that the thymus at young adult human was already definitely decreased in terms of the capacity to provide new T cells for peripheral lymphoid tissues.

As to atrophic thymus of old mice, we examined its function by grafting it into nude mice and found that the old thymus was still maintaining to a certain extent the potential to provide T cells for the periphery. However, T cells emigrated from the old thymus were functionally impaired compared to those from the newborn thymus (Kurashima *et al.*, 1993).

Regarding the atrophic thymus of human adults, controversy exists as to how much function is remaining. Most people believe that atrophic fatty thymuses of the human adult are not working anymore. While, Douek *et al.* (1998) reported that the thymus of human adults were still capable of producing thymocytes.

In this communication, we have tried to present morphological basis to support the concept that atrophic thymuses of human adults and the elderly are still functioning, although low in the level.

*Corresponding author. Tel.: +81-3-5803-5173. Fax: +81-3-3813-1790. E-mail: hirokawa.pth2@med.tmd.ac.jp

ISSN 1740-2522 print/ISSN 1740-2530 online © 2003 Taylor & Francis Ltd
DOI: 10.1080/10446670310001598465

In order to analyze the thymic microenvironment, we employed two monoclonal antibodies (MoAb) which could distinguish between medullary and subcortical thymic epithelial cells, and those within the cortex. Adult thymic tissue is split into fragments in fatty tissue, but we found that these fragments still maintained the basic structure of both cortex and medulla, and thymocytes in those fragments were still actively proliferating and undergoing apoptosis.

### MATERIALS AND METHODS

### Materials

Thymic tissues were obtained from patients undergoing cardiac surgery. The tissues were partly frozen in liquid nitrogen and stored in a deep freezer (−80°C), and partly fixed in formalin for paraffin sections. The patients ranged in age from 2 to 78 years. Thymic tissues were also obtained from autopsy cases of fetus and new born babies who died from heart anomaly, and processed in the same way.

### Immunohistology

For the staining of IL-1R, UH1, CD4 and CD8, frozen sections were used. Cryostat sections were air-dried, washed in phosphate buffered saline (PBS), fixed with acetone, washed again and then incubated for 1 h with primary antibodies. After washing, the sections were incubated for 1 h with either horseradish peroxidase (HRP) conjugated streptavidin (DAKO) or HRP-conjugated secondary antibodies.

For the staining of Ki-67, paraffin sections were used. Deparaffinized sections were first treated with 0.1% trypsin at 37°C for 30 min, washed in PBS and heated in microwave oven at 95°C for 20 min. Then the sections were incubated over night with primary antibodies at 4°C, and incubated with HRP-conjugated secondary antibodies for 1 h at room temperature.

Bound HRP was detected with the substrate containing 3,3′-diaminobenzidine.

### Antibodies

Primary antibodies employed in the present study were monoclonal antibody (MoAb) to mouse IL-1R (Pharmingen, San Diego), MoAb to cytokeratin (COSMO BIO, Japan), biotin-conjugated MoAb to human CD4 (Pharmingen), MoAb to human CD8 (DAKO, Denmark) and MoAb to human thymic cortical epithelial cells (UH-1). UH-1 was produced in our laboratory (Hirokawa *et al.*, 1998b).

Secondary reagents were HRP-conjugated MoAb to mouse immunoglobulins (DAKO), HRP-conjugated MoAb to rabbit antibodies (DAKO), HRP-conjugated streptavidin (DAKO), rhodamine isothiocyanate (RITC)-conjugated antibody to mouse antibodies (DAKO) and fluorescence isothiocyanate (FITC)-conjugated antibody to streptavidin (CALTAG LABORATORIES, USA).

### Immunoblotting

Immunoblotting was performed as described previously (Utsuyama *et al.*, 1997). Briefly, cell lysates were prepared from frozen thymic tissue with lysis buffer [50 mM Tris-HCl (pH 7.5), 1% (W/V) Nonidet P-40, 0.02% NaN3, 1 mM PMSF, 10 μg/ml Aprotinin, 100 μM Leupeptin and 100 μM TPCK]. Cell lysates were subjected to 7.5% sodium dodecyl sulfate (SDS)-polyacrylamide gel electrophoresis (PAGE). The transferred membranes were blocked in 10 × Block Ace (Dainippon Pharmaceutical, Japan) and incubated in relevant primary antibody for 1 h. After washing in Block Ace with 0.05% tween 20, the membranes were incubated in HRP-conjugated anti-rat immunoglobulins (DAKO, Denmark). Bands in the washed membrane were detected by a chemiluminescence reagent system (Amersham).

### Detection of Apoptotic Cells

To determine apoptotic cells, terminal deoxy-transferase (TdT)-mediated dUTP nick end labeling (TUNEL) method was used as described previously (Ishiyama *et al.*, 1999). Briefly, sections were air-dried, fixed in 4% paraformaldehyde (PBS pH 7.4) at room temperature for 30 min and incubated with 0.1% Triton X-100 diluted in 0.1% sodium citrate on ice for 2 min. After washing, TdT, fluorescein isothiocyanate (FITC)-dUTP and dATP (Boehringer Mannheim Biochemica, Mannheim, Germany) were applied to the sections, which were then incubated in a moist chamber at 37°C for 60 min. Horseradish-peroxidase (HRP)-conjugated antibody specific for fluorescein (POD converter, Boehringer Mannheim) was employed for detecting FITC-dUTP labeling. Bound HRP was detected with the substrate containing 3,3-diaminobenzidine. As negative controls, reactions were performed in the absence of TdT.

### Counting of Immunohistologically Positive Cells

The population density of Ki-67 positive cells and apoptotic cells were determined by counting the number of positive cells in 1000 cells under light microscopy.

### Statistics

Student's *t*-test was used to determine statistical difference in values between two groups.

### RESULTS

### Search for Antibodies distinguishing Medullary Thymic Epithelial Cells from Cortical Ones

In order to analyze thymic microenvironment, markers distinguishing between cortical and medullary thymic epithelial cells were indispensable. During immunohistological staining of mouse thymus by various monoclonal

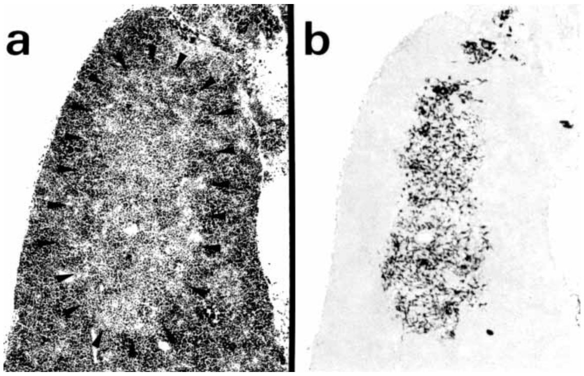

FIGURE 1 Thymus of 4 weeks old C57BL/6 mouse, stained with Hematoxylin-Eosin (a) and immunohistological staining with monoclonal antibody (MoAb) to murine IL-1R type I (b). IL-1R type I was strongly expressed in medullary thymic epithelial cells. Medulla, surrounded by arrows in (a) (Magnification, × 100).

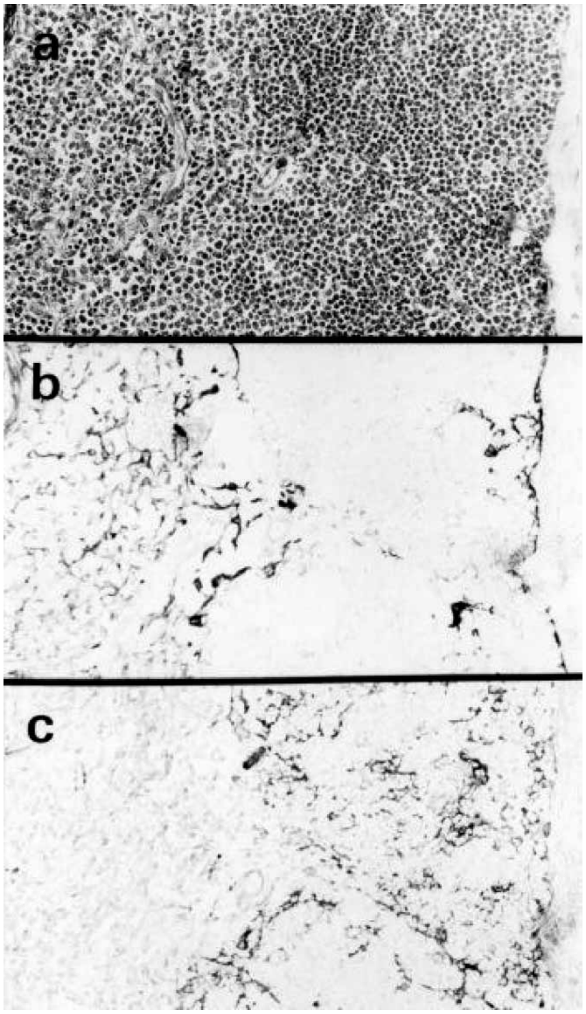

FIGURE 2 Human thymus of 4 years old infant, frozen section. (a), Hematoxylin-eosin stain. (b), Stain with anti-murine IL-1R MoAb. (c), Stain with UH-1 MoAb. IL-1R was expressed in medullary epithelial cells and subcapsular cortical ones. UH-1 was expressed in epithelial cells within the cortex. (Magnification, × 100).

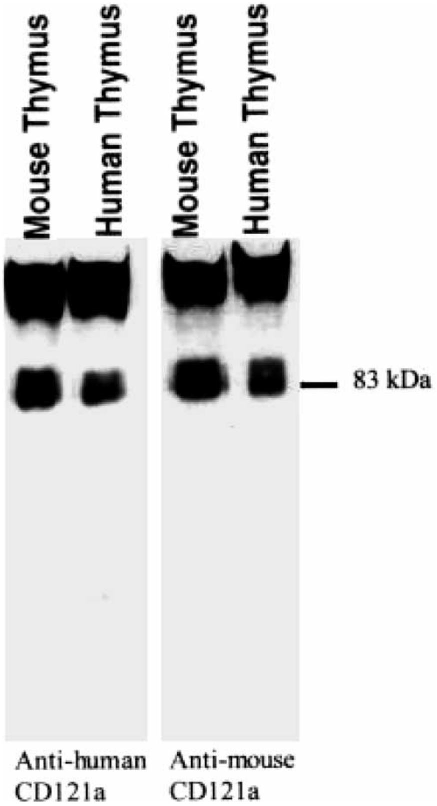

FIGURE 3 Immunoblotting of mouse and human thymus by using MoAb to human and murine IL-1R (CD121a). After immunoprecipitation of human and mouse thymic lysates with anti-mouse IL-1R Ab, immunoblotting were performed with anti-mouse or anti-human IL-1R Ab. The anti-mouse IL-1R antibody recognized both human as well as mouse IL-1R (CD121a) at about 83 kDa.

antibodies (MoAb), we incidentally found that IL-1R type I was strongly expressed in medullary thymic epithelial cells (Fig. 1). IL-1 is a multifunctional cytokine binding to various types of cells and the receptor for IL-1 is known to be expressed in many types of cells such as T cells, B cells, fibroblasts and keratinocytes. By routine immunohistological method, the expression of IL-1R type I was detected only in medullary epithelial cells of mouse thymus.

Using the same MoAb, we examined the expression of IL-1R type I in human infant thymus and found that it was expressed in medullary epithelial cells and subcapsular cortical ones, but not in epithelial cells within the cortex (Fig. 2a,b).

UH-1 is a monoclonal antibody, which was produced in our laboratory. It specifically binds to thymic epithelial cells within the cortex, but neither to medullary nor subcapsular epithelial cells (Fig. 2c). By fluorescent immunohistological examination using two colors, the localization of IL-1R positive cells was definitely different from UH-1 positive cells (Fig. 6a).

Then, we performed immunoblotting to confirm that the MoAb really recognized human IL-1R as well as mouse IL-1R. The result (Fig. 3) indicated that the band of similar size was detected in both mouse and human thymus.

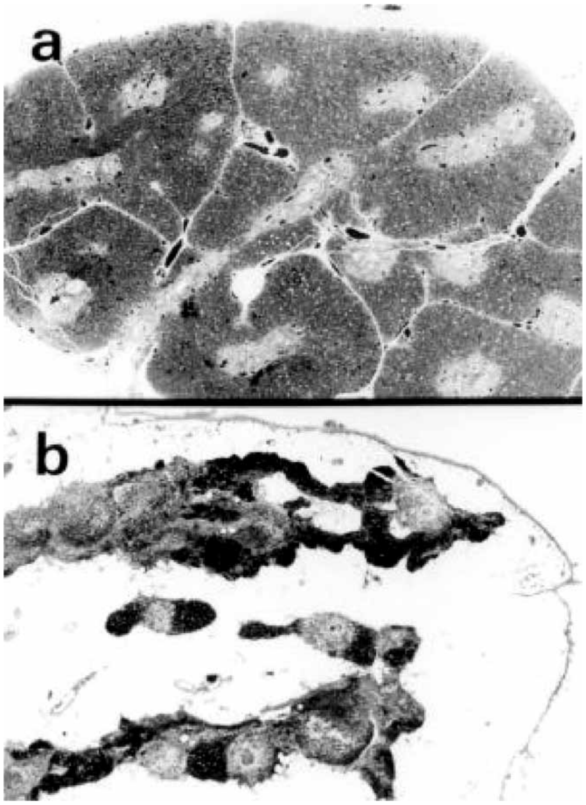

FIGURE 4 The thymus from human fetus (38 weeks) (a) and 28-year-old adult (b) stained with Hematoxylin-eosin (Paraffin section). The adult thymus is prominently infiltrated by fat, and the volume of actual thymic tissue is reduced, and scattered in the fatty tissue. (Magnification, × 12.5).

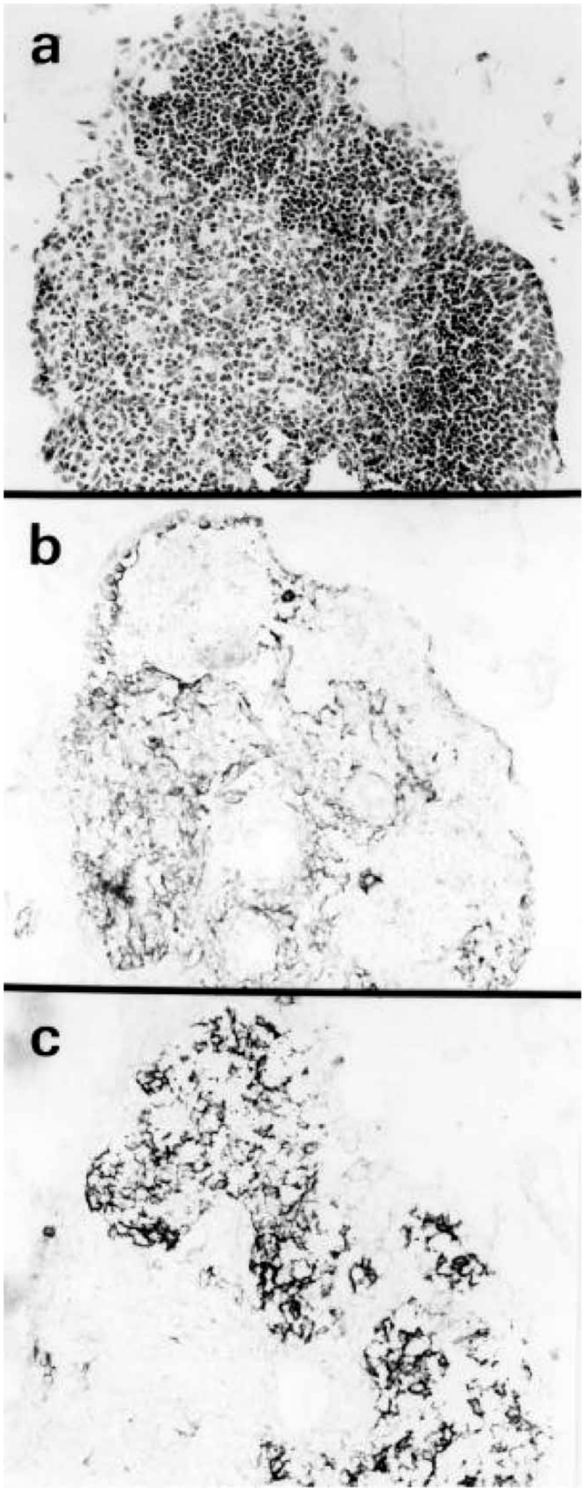

FIGURE 5 A lymph follicle-like area in thymus of 56-year-old male. Serial frozen sections, stained with Hematoxylin-eosin (a), with anti-IL-1R MoAb (b), with UH-1 MoAb (c). Areas positive for anti-IL-1R MoAb are negative for UH-1 MoAb. (Magnification, × 200).

Thus, we concluded that IL-1R could be used a marker of thymic epithelial cells of the medulla and of the subcapsular layer covering cortex, and UH-1 could be used as a marker of thymic epithelial cells within the cortex.

### Age-related Changes of the Microenvironment of Human Thymus

The basic structure of thymus is composed of cortex and medulla and is clearly observed in the newborn baby (Fig. 4a). However, it is completely lost in the thymus of adults over 20 years of age, due to progressive infiltration of fatty tissue (Fig. 4b). The adult thymus appears to be composed of two different areas; i.e. one is lymph follicle-like portion, rich in lymphocytes and the other pale portion, poor in lymphocytes. Using UH-1 and MoAb to IL-1R, we examined lymph follicle-like areas of adult thymus (Fig. 5a–c). We found IL-1R was positive in the area where lymphocytes were poor in number and in the thymic epithelial cells covering lymph follicle-like area (Fig. 5b). On the contrary, UH-1 was positive in the thymic epithelial cells within the lymph follicle-like area (Fig. 5c). The structural relation between these two portions was clearly seen in two-colored fluorescent pictures (Fig. 6). In adult thymus, the red-stained lymph follicle-like area (C) was surrounded by green-stained epithelial cells (Fig. 6b). These findings suggested that the lymph follicle-like areas of adult thymus could be similar to the cortex of infant thymus and the areas positive for

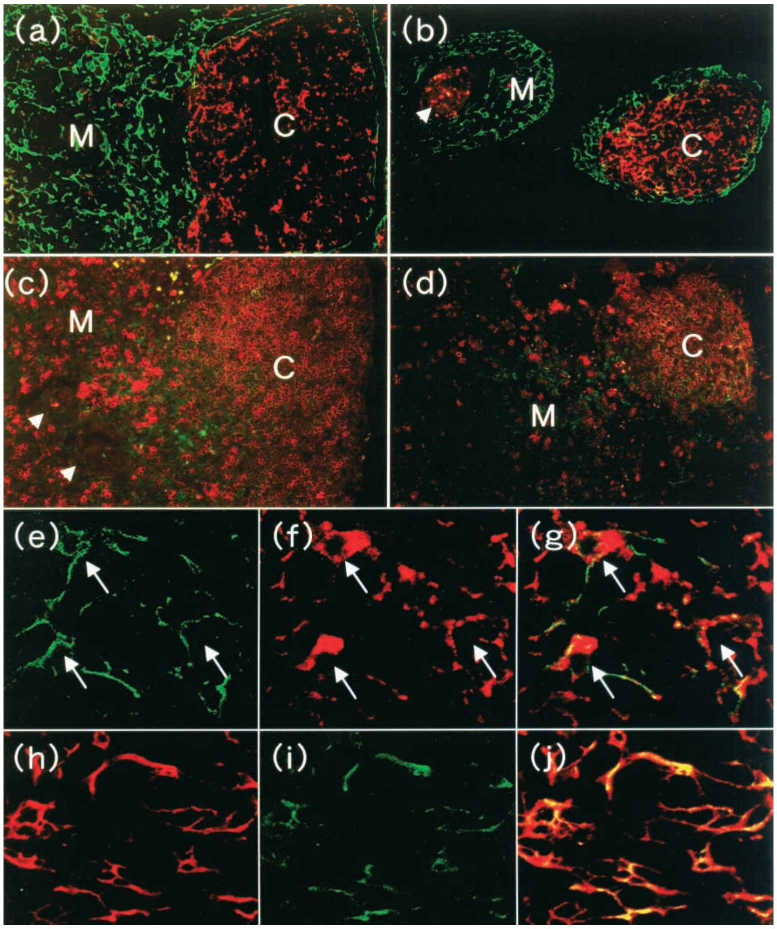

FIGURE 6 Two colors-fluorescent immunohistological examination of thymus from 4 years old infant (a,c) and adult (b,d). Thymic epithelial cells stained with anti-IL-1R MoAb were green (FITC) and those with UH-1 MoAb were Red (RITC). The localization of IL-1R positive cells was definitely different from UH-1 positive cells (a,b). Lymphocytes are stained with anti-CD4 MoAb (green, FITC) and anti-CD8 MoAb (red, RITC). The lymphocytes in the cortex (C) and lymph follicle-like area are mostly $CD4^+8^+$ double positive cells (yellowish colour). But the lymphocytes in the medulla (M) are mostly single positive, either $CD4^+$ or $CD8^+$. Arrows indicate Hassall's corpuscles. Two colors-fluorescent immunohistological staining of infant thymus with anti-keratin antibody (e, green), UH-1 antibody (f, red) and double colors (g). Arrows indicate co-localization of keratin and UH-1. Two colors immunohistological staining of infant thymus with anti-keratin antibody (h, red), anti-IL-1R antibody (I, green) and double colors (j). Almost complete co-localization of keratin and IL-1R is observed.

IL-1R could be similar to medullary and subcapsular epithelial cells. Then, we examined characteristics of lymphocytes in these areas.

### Proliferation and Apoptosis of Thymocytes in Human Thymus of Various Ages

We found that lymphocytes of lymph follicle-like areas were composed of $CD4^+CD8^+$ double positive thymocytes as observed in the cortex of infant thymus (Fig. 6c,d). On the contrary, lymphocytes in the IL-1R positive areas were composed of single positive cells, either $CD4^+$ or $CD8^+$ (Fig. 6c,d).

Then we examined proliferative activity of lymphocytes in thymus of infant (Fig. 7a) and adult (Fig. 7b) by using Ki-67 antibody. Many of thymocytes in the cortex of infant thymus (Fig. 7c) and lymph follicle-like areas of adult thymus (Fig. 7d) were positive for Ki-67. Ki-$67^+$ cells were not observed in the medulla of infant thymus. As the density of Ki-67 positive cells appeared to be lower in adult thymus as compared with infant thymus, we examined 25 thymuses at various age in terms of density of Ki-67 positive cells. We found that the density significantly decreased with advance of age (Fig. 8).

Because of selection process during T cell differentiation in the thymus, apoptosis was commonly found in the cortex of infant thymus (Fig. 7e). Apoptosis was also found in the lymph follicle-like areas of adult thymus (Fig. 7f). Immunohistologically, the density of apoptosis appeared to be higher in the lymph follicle-like areas of adult thymus than in the cortex of infant thymus. We compared 7 cases of infant thymus and 8 cases of adult thymus in terms of density of apoptosis and found that density of apoptosis significantly increased in the latter (Fig. 9).

The present paper revealed that (1) basic thymic microenvironment of cortex and medulla was preserved in the adult and aged thymus; (2) lymphocytes in the lymph follicle-like areas were mostly $CD4^+CD8^+$ cells and many of them were in proliferative phase; (3) density of

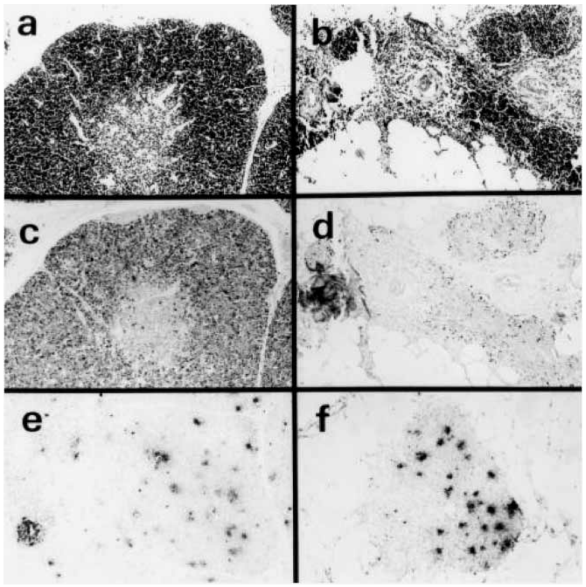

FIGURE 7 Proliferation and apoptosis of lymphocytes in thymus of fetus (38 weeks) (a,c), infant (e) and adult (41-year-old, b,d). Hematoxylin-eosin stain of fetal (a), and adult thymus (b). Immunohistological staining with Ki-67 of fetal (c), and adult thymus (d). Positive cells are mainly present in the cortex of fetal thymus and lymph follicle-like areas of adult thymus. Few cells are positive in the medulla (c,d). Apoptosis, detected with TUNEL method in the thymus of 4 years old infant (e) and 56 years old adult (f). The density of apoptosis appeared to be higher in the lymph follicle-like areas of adult thymus than in the infant thymus. (a,b,c,d are paraffin section. e,f are frozen section. Magnification, $\times 200$).

proliferating cells decreased in the lymph follicle-like areas with age; (4) density of apoptosis in these areas increased with age; (5) these findings taken together supported the concept that thymopoiesis was ongoing in adult and aged thymus, although the magnitude greatly diminished.

### DISCUSSION

Mouse thymus quickly grows after birth and peaks in weight around at 4 to 6 weeks of age. From functional viewpoint, the thymic activity to promote T cell differentiation was the highest at newborn stage and quickly declined thereafter. Actually, the functional capacity of mouse thymus at 4 weeks of age maintained only 6% of newborn level in terms of provision of T cells to the periphery (Hirokawa *et al.*, 1994).

Thymus of the aged mice becomes atrophic, but still preserves the basic structure of cortex and medulla. When atrophic thymus of the aged mice is grafted into nude mice, it shows a significant level of the activity to promote T cell differentiation, although immunological functions of these T cells apparently lower as compared with T cells produced by young thymus (Hirokawa *et al.*, 1982).

Composition of T cell subsets changes with age in mouse as well as human; i.e. an increase of memory T cells with a concomitant decrease of naive T cells

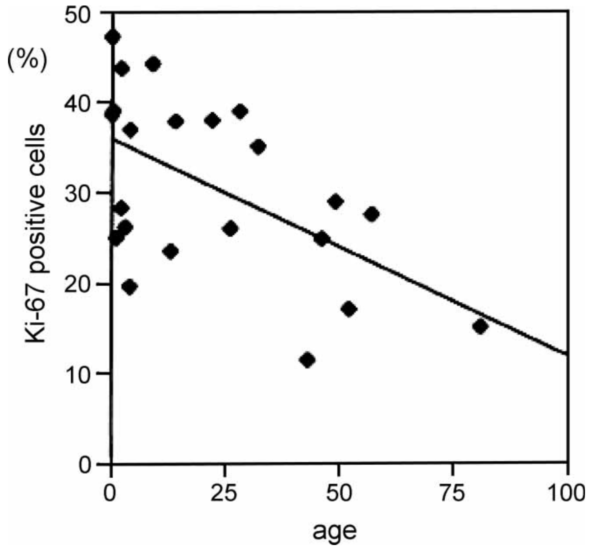

FIGURE 8 The decrease of Ki-67 positive cells in the cortex with advance of years. $y = -0.242x + 35.981$, $r = 0.565$, $p < 0.05$.

(Utsuyama *et al.*, 1991). The change in T cell subsets in the peripheral lymphoid tissues in mouse can be partly ascribed to the age-related change of thymic function (Kurashima *et al.*, 1993).

In human, information on functional aspect of adult and elderly thymus has been limited. From morphological viewpoint, fatty infiltration was observed in the thymus of as early as 5 years old (Hirokawa *et al.*, 1985). Furthermore, precise histopathological examination of human thymus of various ages revealed that the reduction

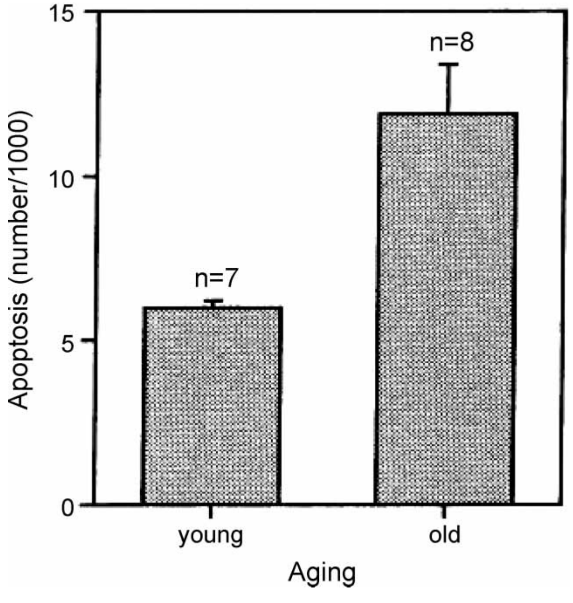

FIGURE 9 The density of apoptosis, higher in the lymph follicle-like areas of adult thymus than in the cortex of infant thymus. Results were analyzed using Student's *t*-test ($p = 0.0061$). Infant thymus, 7 cases (age; $3.5 \pm 0.9$) and adult thymus 8 cases (age; $61.0 \pm 1.9$).

in thymic parenchyma starts as early as one year of age (Steinmann *et al.*, 1985). George and Ritter (1996) reported that the thymic involution might be an evolutionary outcome that reflects good housekeeping on the part of our ancestors.

MacKall *et al.* (1995) reported functional aspect of human adult thymus. They examined the regeneration of T cells in cancer patients after intensive chemotherapy and found that a significant decline of thymic function was observed in patients over 20 years of age. Regarding atrophic thymus of human adults, Doueck *et al.* (1998) reported that the thymuses of human adults were still capable of producing thymocytes, actively rearranging TCR genes and provide newly T cells for the peripheral lymphoid tissues.

Thus, in this paper, we aimed to obtain morphological evidence to support that atrophic human thymus of the adult and elderly is actually functioning. For this purpose, we explored tools for analysis of the thymic microenvironment.

In a previous report Kuwata *et al.* (1995) we reported that protein tyrosine kinases of receptor type were differentially expressed in thymic epithelial cells of cortical and medullary type; i.e. bek and met were expressed in medullary type, and flg in cortical type. According to this finding, we examined expression of these proteins in human thymic tissue, but differential expression was not clearly observed (unpublished data).

We finally found that two monoclonal antibodies, IL-1R and UH-1 were useful to distinguish between cortical and medullary epithelial cells in human thymus. In this regard, Deyerle *et al.* (1992) reported that lymphoid tissues in healthy animals expressed low or non-detectable levels of receptor transcript, whereas non-lymphoid tissues constitutively expressed readily detectable levels of IL-1R mRNA, although they did not make mention of thymus.

In the present paper, we found that IL-1R was expressed in thymic epithelial cells of medulla and subcapsular region, and UH-1 was expressed in thymic epithelial cells within the cortex of infant thymus. Both IL-1R positive and UH-1 positive regions were well preserved and differentially expressed in fragments scattered in fatty tissue of human adult and elderly thymus. Moreover, we found that proliferation of thymocytes was ongoing together with apoptosis in UH-1 positive regions, which were rich in lymphocytes. It was of interest to note that proliferative capacity of thymocytes significantly decreased, while apoptosis increased with age.

Therefore, we have concluded that the thymic fragments are not simple fragments but could be referred to as "thymic units" which are composed of cortical and medullary portions, and actually capable of providing a certain number of T cells even in young and old adults.

Questions to be solved are functional role of IL-1R expressed on thymic epithelial cells and molecular determination of antigen recognized by UH-1 MoAb. Experiments are now ongoing and results will be reported later.

### References

Deyerle, K., Sims, J.E., Dower, S.K. and Bothwell, M.A. (1992) "Pattern of IL-1 receptor gene expression suggests role in non-inflammatory process", *J. Immunol.* **149**, 1657.

Douek, D.C., McFarland, R.D., Keiser, P.H., Sullivan, J.L., Jamieson, B.D., Zack, J.A., Picker, L.J. and Koup, R.A. (1998) "Changes in thymic function with age and during the treatment of HIV infection", *Nature* **396**, 690.

George, A.J.T. and Ritter, M.A. (1996) "Thymic involution with ageing: obsolescence or good housekeeping?", *Immunol. Today* **17**, 267.

Hirokawa, K. (1985) "Autoimmunity and aging", In: Cruse, J.M. and Lewis, R.E., Jr, eds, Autoimmunity: Basic Concepts; Systemic and Selected Organ-specific Diseases, p 251.

Hirokawa, K. (1998) "Immunity and ageing", In: Pathy, M.S.J., ed, Principles and Practice of Geriatric Medicine (John Wiley and Sons Ltd), p 35.

Hirokawa, K. and Makinodan, T. (1975) "Thymic involution: effect on T cell differentiation", *J. Immunol.* **114**, 1659.

Hirokawa, K., Sato, K. and Makinodan, T. (1982) "Influence of age of thymic graft on the differentiation of T cells in nude mice", *Clin. Immunol. Immnopathol.* **24**, 251.

Hirokawa, K., Utsuyama, M., Katsura, Y. and Sado, T. (1988) "Influence of age on the proliferation and peripheralization of thymic T cells", *Arch. Pathol. Lab. Med.* **112**, 12.

Hirokawa, K., Utsuyama, M., Hashimoto, T., Masaoka, A. and Goldstein, A.L. (1988) "Immunohistochemical studies in human thymomas. Localization of thymosin and various cell markers", *Virchow Arch. B Cell. Pathol.* **55**, 371.

Hirokawa, K., Utsuyama, M., Kasai, M., *et al.* (1994) "Understanding the mechanism of the age change of thymic involution to promote T cell differentiation", *Immunol. Lett.* **40**, 269.

Ishiyama, N., Utsuyama, M., Kitagawa, M. and Hirokawa, K. (1999) "Immunological enhancement with a low dose of cyclophosphamide in the aged mice", *Mech. Ageing Dev.* **111**, 1.

Kurashima, C., Utsuyama, M. and Kasai, M. (1993) "The role of thymus in aging of Th cells subpopulations and age-associated alteration of cytokine production of T cells in mice", *Int. Immunol.* **7**, 1177.

Kuwata, T., Takahashi, H., Kasai, M., Kurashima, C., Utsuyama, M. and Hirokawa, K. (1995) "Differential expression of protein tyrosine kinases in the cortical and medullary thymic epithelial cell lines", *Biochem. Biophys Res. Comm.* **213**, 768.

MacKall, C.L., Fleisher, T.A., Brown, M.K., *et al.* (1995) "Age, thymopoiesis and $CD4^+$ T lymphocytes regeneration after intensive chemotherapy", *N. Engl. J. Med.* **332**, 143.

Steinmann, G.G., Klaus, B. and Muller-Hermelink, H-K. (1985) "The involution of the ageing human thymic epithelium is independent of puberty. A morphometric study", *Scand. J. Immunol.* **22**, 563.

Utsuyama, M., Hirokawa, K., Kurashima, C., Fukayama, M., Inamatsu, T., Suzuki, K., Hashimoto, W. and Sato, K. (1991) "Differential age-change in the number of $CD4^+CD45RA^+$ T and $CD4^+CD29^+$ T cells in the human peripheral blood", *Mech. Ageing Dev.* **63**, 57.

Utsuyama, M., Wakikawa, A., Tamura, T., Nariuchi, H. and Hirokawa, K. (1997) "Impairment of signal transduction in T cells from old mice", *Mech. Ageing Dev.* **93**, 131.

